# Wireless-Powered Chemical Sensor by 2.4 GHz Wi-Fi Energy-Harvesting Metamaterial

**DOI:** 10.3390/mi10010012

**Published:** 2018-12-25

**Authors:** Wonwoo Lee, Yonghee Jung, Hyunseung Jung, Chulhun Seo, Hosung Choo, Hojin Lee

**Affiliations:** 1 Department of ICMC Convergence Technology, Soongsil University, Seoul 06978, Korea; melanie_lee@ssu.ac.kr (W.L.); wjddydg@ssu.ac.kr (Y.J.); 2 School of Electronic Engineering, Soongsil University, Seoul 06978, Korea; jhs0070@ssu.ac.kr (H.J.); chulhun@ssu.ac.kr (C.S.); 3 School of Electronic and Electrical Engineering, Hongik University, Seoul 04066, Korea; hschoo@hongik.ac.kr

**Keywords:** energy-harvesting metamaterial, wireless chemical sensor, metamaterial sensor

## Abstract

Metamaterial Sensors show significant potential for applications ranging from hazardous chemical detection to biochemical analysis with high-quality sensing properties. However, they require additional measurement systems to analyze the resonance spectrum in real time, making it difficult to use them as a compact and portable sensor system. Herein, we present a novel wireless-powered chemical sensing system by using energy-harvesting metamaterials at microwave frequencies. In contrast to previous studies, the proposed metamaterial sensor utilizes its harvested energy as an intuitive sensing indicator without complicated measurement systems. As the spectral energy-harvesting rate of the proposed metamaterial sensor can be varied by changing the chemical components and their mixtures, we can directly distinguish the chemical species by analyzing the resulting output power levels. Moreover, by using a 2.4 GHz Wi-Fi source, we experimentally realize a prototype chemical sensor system that wirelessly harvests the energy varying from 0 mW up to 7 mW depending on the chemical concentration of the water-based binary mixtures.

## 1. Introduction

Metamaterials are artificially engineered structures that show exotic electromagnetic properties including strong field enhancement and localization of the incident waves [1,2], which consequently offer high-quality resonances and sensing abilities [3,4,5]. With these advantages, metamaterial-based sensor systems have attracted considerable attention for highly sensitive and nondestructive detection applications such as temperature [6], food quality [7,8,9], humidity [10], chemical [11,12,13,14,15,16], and biological sensor systems [17,18,19,20,21]. In particular, metamaterial-based chemical sensor systems are increasingly necessary for use as an environment sensor where immediate and accurate detection of various chemical substances is required for human safety. However, in real-time applications, most of the conventional metamaterial-based sensor systems are limited by the requirement of external equipments such as a network analyzer [14,15,16], spectroscopy systems [9,16,17,18,19], and liquid pump systems [12,13,14,15]. 

To overcome these problems, energy-harvesting metamaterials can be a good candidate for a new sensing platform because their spectral resonance properties can be extracted from the harvested energies. Energy-harvesting metamaterials have been recently studied for the conversion of external electromagnetic energy into a direct current (DC) signal to supply electric power to diverse wireless power systems [22,23,24,25,26] and wireless sensor networks [27,28,29]. As the resonance properties of the metamaterials are sensitive to their environment [15,17,18], we can expect that the maximum harvesting rate can be sensitively changed by various chemical substances dropped on the energy harvesting metamaterials. Therefore, by plotting the variation of the energy harvested from the specific incident waves, the change in the surrounding environment owing to the change in the concentration or species of chemical substances can be analyzed.

In this paper, we proposed a novel self-powered energy-harvesting metamaterial sensor system operating at microwave frequencies. A commercially available 2.4 GHz Wi-Fi router with an antenna was used as a power source to supply electromagnetic waves wirelessly to our metamaterial sensor. In our sensor design, a micro-channel was constructed at the gap of the metamaterial pattern to confine the chemicals in the area of the enhanced electric field to effectively detect minute amounts of chemicals [15]. The chemical components of the various solutions could be identified by the metamaterial-based sensor through the analysis of the energy-harvesting rate of our sensor system, which was varied by changing the concentration and species of chemicals. In contrast to the previously reported metamaterial sensor systems, by converting the harvested free microwave energy into DC current, the proposed metamaterial sensor could exhibit satisfactory sensing results with simple LED indicators without requiring complicated measurement setup and additional input energy sources.

## 2. Materials and Methods

### 2.1. Design of Energy-Harvesting Metamaterial Sensor

**Figure 1:**
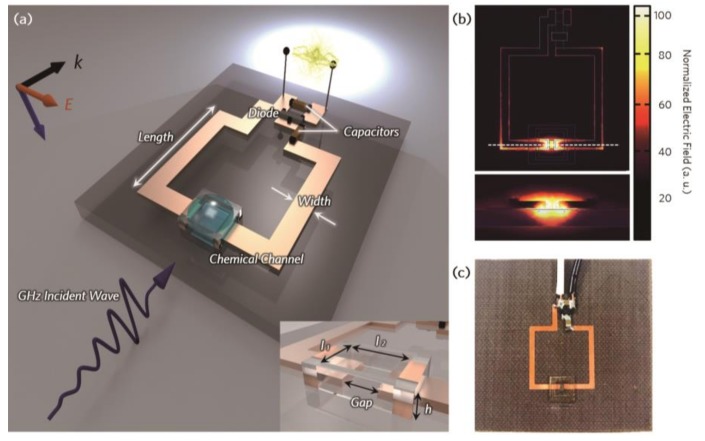
Schematics of the proposed system and design. (**a**) Schematic of the proposed energy-harvesting metamaterial sensor system. (**b**) Top and cross-sectional views of the simulated electric field distribution of the proposed metamaterial device at the resonance frequency. (**c**) Photograph of the fabricated metamaterial sensor system.

Figure 1a illustrates the schematic view of the proposed energy-harvesting metamaterial sensor, which consists of a single split-ring resonator (SRR), chemical channel, and rectifier circuit for converting the incident microwave into DC output voltage. As the SRR structure can be considered an LC circuit consisting of an inductive metallic loop and capacitive gap, an electric field is strongly induced only at the SRR gap area at the resonance frequency, as shown in Figure 1b. Hence, if the chemical components are captured within the channel placed in this gap, the resonance frequency of the metamaterials can be effectively changed, because the electrical permittivity of the chemical components affects the effective capacitance around the gap of the SRR resonator. Simultaneously, to operate our metamaterial structure as an energy-harvesting device, a Greinacher voltage doubler circuit [22] was adopted and connected to two output nodes of SRR to rectify the induced current in the SRR structure by the incident microwave.

A geometrical structure of the metamaterial sensor was designed to operate in the range approximately 2 GHz to 3 GHz with the resonance frequency of 2.4 GHz using ANSYS high-frequency structure simulator (HFSS). An SRR made of copper pattern was fabricated on a Taconic TLC substrate with the permittivity of ε = 3.2 using photolithography. Based on the simulation results, the geometry of the SRR was optimized to have the length (*l*) of 13 mm, width (*w*) of 1 mm, and gap (*g*) of 1 mm. The rectifier circuit was connected to the electrodes of the SRR loop. The chemical channel was made of SU-8 photoresist owing to its good chemical resistance [30,31].

### 2.2. Sample Fabrication

The proposed energy-harvesting metamaterial chemical sensor was fabricated by the following process. First, a 35-μm-thick copper on 0.78-mm-thick Taconic TLC substrate was patterned using photolithography process for single split-ring resonator (SRR) pattern. After patterning SRR, to fabricate chemical channel, SU-8 100 photoresist (Microchem Corporation, Westborough, MA, USA) was spin-coated on copper-patterned substrate and soft-baked at 70 °C for 5 min followed by 100 °C for 100 min. Then, the photoresist-coated substrate was exposed to UV aligner using photomask. After exposure, the substrate was annealed at 70 °C for 1 min followed by 100 °C for 35 min. Then, by dissolving the substrate into the SU-8 developer (Microchem Corporation), the chemical channel was successfully formed. Finally, for the rectifier circuit, the diode (HSMS-2862-BLGK, San Jose, CA, USA) and chip capacitors were connected by soldering. As shown in Figure 1c, the chemical channel was formed at the SRR gap to detect the chemical components by using a simple drop casting method. The chemical channel was designed with the dimensions *l*_1_ = 300 μm, *l*_2_ = 500 μm, and *h* = 600 μm. The fabricated metamaterial sensor device is shown in Figure 1c with a transparent chemical channel covering the SRR gap area.

### 2.3. Water-based Bianry Mixture Chemical Components Preparation

Water-based binary mixture chemical components were prepared by mixing deionized water (DI water) with solution-state chemicals such as toluene, chloroform, ethanol, acetone, and methanol with a purity of 99.5% or higher. To control the exact concentration and the precise amount of chemicals, a micropipette was used to extract DI water and chemicals. For the calculation of concentration, volume percent (*v/v* % chemical) was used. For example, in order to get 100 mL acetone chemical components with 20% concentration, 20 mL of acetone solution was dissolved in 80 mL DI water solution.

## 3. Results

### 3.1. Spectral Resonance Frequency Shift of Energy-Harvesting Metamaterial Sensor

**Figure 2:**
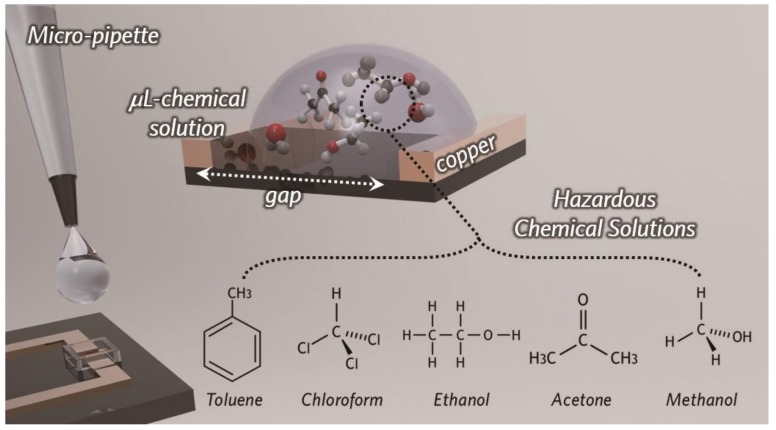
Schematic of sample preparation by drop casting and structures of sensing chemical materials.

Figure 2 shows the schematics of sample preparation by drop casting and structures of sensing chemical materials. Chemical components are dropped into the micro-channel using micropipette to accurately control the amounts of chemicals. Since this micro-channel around the SRR gap minimizes geometrical variation factors of the liquid chemical components, we can evaluate that the resonance changes of our metamaterial sensors solely originated from the dielectric constant of the chemical components.

**Figure 3:**
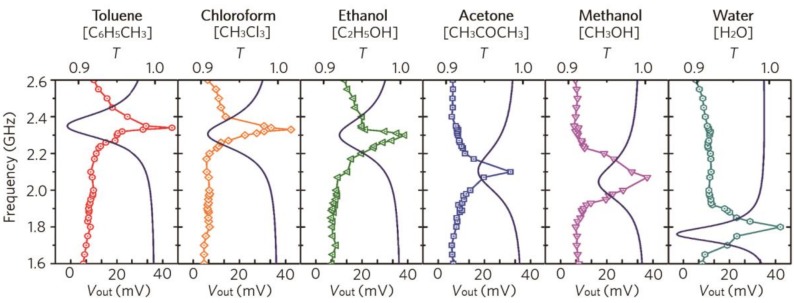
Simulated transmission spectra (line) and measured output voltage (*V*_out_) spectral (symbol) of the proposed metamaterial sensor system for various chemical substances.

To determine how effectively the chemical substance at the SRR gap affected the spectral resonance of our metamaterial sensor, we simulated the transmission spectra of the metamaterial sensor for various chemical substances with different dielectric constants, and confirmed that the resonance frequency of transmission of the metamaterial sensor was shifted by the use of different species of chemical components having different dielectric constants as shown in Figure 3. To verify the simulation results, we filled the chemical channel of the metamaterial sensor with various chemical substances such as toluene (ε_t_ = 2.38) [32], chloroform (ε_c_ = 4.81) [33], ethanol (ε_e_ = 9) [34], acetone *(*ε_a_ = 21) [35], methanol (ε_m_ = 33.1) [34], and water (ε_w_ = 79.5) [34], and measured the output voltage (V_out_) of the metamaterial sensor in the frequency domain. To measure the spectral resonance properties of the energy-harvesting metamaterial sensor, a horn antenna was connected to the signal generator to generate an incident electromagnetic wave and the metamaterial sensor was placed at a distance of 10 cm from the horn antenna. Then, spectral output voltages and resonance properties [36,37,38] of the proposed metamaterial sensor were measured for different frequencies of the incident wave ranging from 1.8 GHz to 2.7 GHz and all of the measurement system was surrounded by anechoic materials. As shown in Figure 3, the measured output voltage showed distinct resonance frequency shifts for the various chemical components as confirmed by the simulation results. When toluene, chloroform, ethanol, acetone, methanol, and water were captured at the chemical channel, the resonance peak of the metamaterial sensor was shifted to 2.32, 2.28, 2.20, 2.08, 1.97, and 1.78 GHz, respectively.

**Figure 4:**
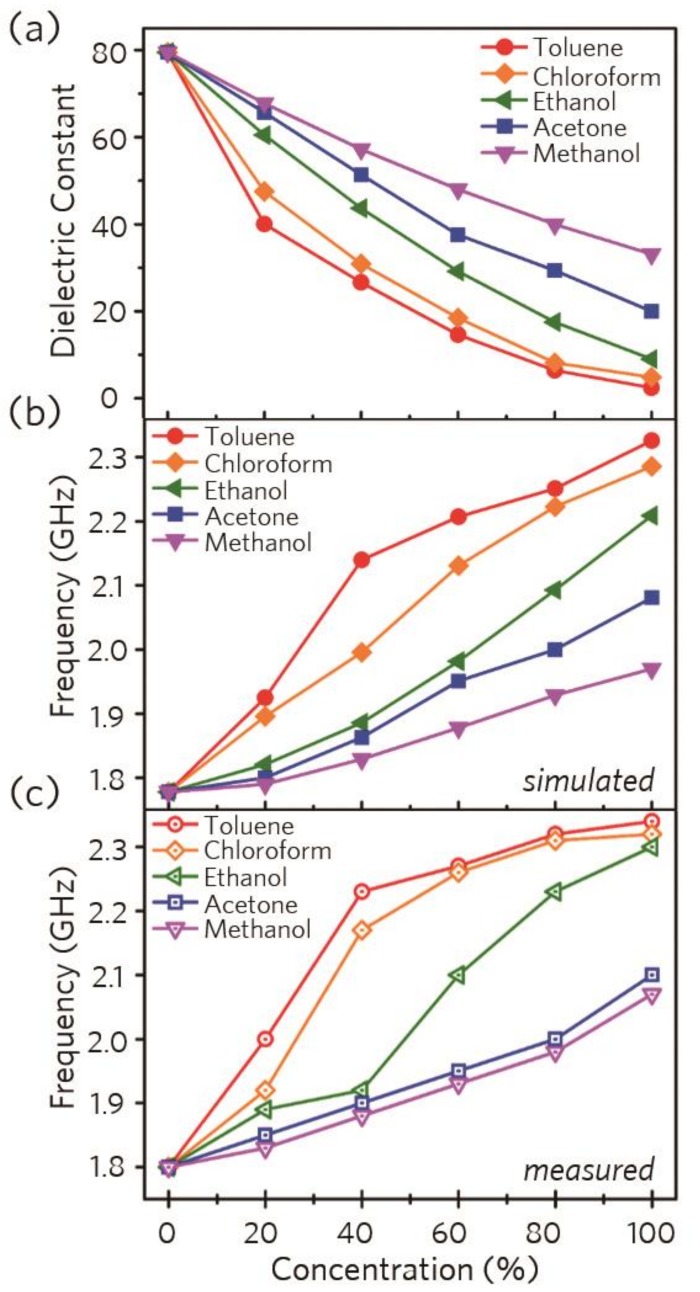
(**a**) Calculated effective dielectric constants for the chemical mixtures. (**b**) Simulated and (**c**) measured resonance frequencies for the chemical mixtures.

To investigate the chemical sensing properties of our proposed system, we experimentally evaluated various chemical mixtures, as shown in Figure 4. As the effective dielectric constant for chemical mixtures can be calculated from the Braggman Equation [39], we plotted the calculated effective dielectric constant for binary chemical mixtures, as shown in Figure 4a. Owing to the discrepancy of the dielectric constant between water and chemical mixtures, the effective dielectric constants can be varied by changing the chemical mixture ratios and concentrations. Based on the calculated effective dielectric constants, we performed a simulation to observe the change in the resonance frequency and confirmed that the resonance frequency shifted sensitively as the concentration changed. An experiment was performed to demonstrate this, and results similar to the simulation results were obtained. Therefore, as shown in Figure 4b,c, the simulated and measured resonance frequencies of the metamaterial sensor were gradually blue-shifted in accordance with the increase in the chemical concentration in the water-based binary mixtures.

### 3.2. Wi-Fi Energy-Harvesting Metamaterial Sensor System

**Figure 5:**
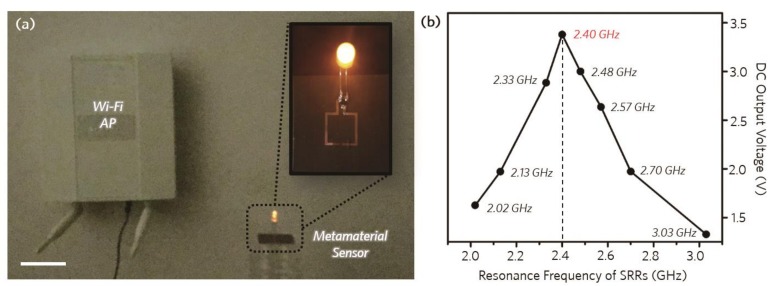
Wi-Fi energy-harvesting sensor system. (**a**) Photograph of the experimental setup for the Wi-Fi energy-harvesting sensor sys-tem. The inset shows the energy-harvesting sensor connected to an LED. (scale bar: 5 cm) (**b**) Measured DC output voltage of the Wi-Fi harvesting metamaterials with different resonance frequencies.

**Figure 6:**
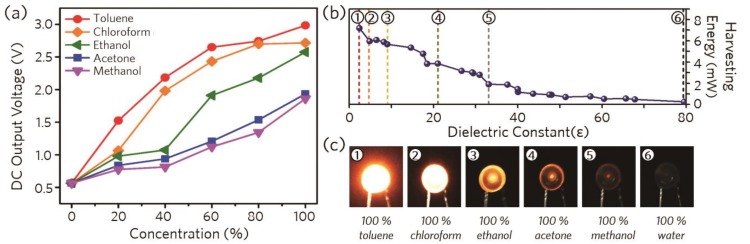
Experimental results for various chemical mixtures. (**a**) Measured DC output voltage of the Wi-Fi harvesting sensor for various concentrations of the chemical mixtures. (**b**) Measured harvesting energy and (**c**) corresponding brightness of the sensor for different dielectric constants of the chemical mixtures.

For practical uses, we adopted an easily accessible and widespread 2.4 GHz Wi-Fi access-point (AP) as an electromagnetic single wave source for our wireless sensing system. The Wi-Fi AP was placed at a distance of 10 cm from the metamaterial sensor and a light-emitting diode (LED) was connected to the metamaterial sensor as an indicator to evaluate the energy-harvesting rate, as shown in Figure 5a. To compare the energy-harvesting rate depending on the resonance properties, we fabricated several devices with different resonance frequencies ranging from 2 GHz to 3 GHz and measured the DC output voltage of the sensors, which indicates the energy harvested from the Wi-Fi sources. As shown in Figure 5b, the highest output voltage of 3.38 V was obtained for the metamaterial device with the resonance frequency of 2.4 GHz, because the resonance frequency of the sensor was matched with the Wi-Fi source frequency. The energy-harvesting rate decreased to 1.5 V when the resonance frequency of the metamaterial sensor shifted to lower or higher frequencies from 2.4 GHz. Thus, we could confirm that the difference in the energy-harvesting rate was due to the discrepancy between the resonance frequencies of the Wi-Fi source and the metamaterial sensor. Hence, by using the resonance frequency shift of the metamaterial sensor for the different chemicals, we can identify the chemical substances and mixture ratios by plotting the variation of the energy-harvesting rate of the metamaterial sensor. Figure 6a shows the measured output voltages of various chemical mixtures with concentrations varying from 0 % to 100 % in increments of 20 %. The maximum output DC voltage of 2.98 V could be obtained for 100 % toluene when the resonance frequency of the device was 2.34 GHz as shown in Figure 6a. When the ratio of water in the chemical mixtures was increased, the output voltage decreased and the minimum output voltage was measured at around 560 mV for 100 % water. To evaluate the chemical sensitivity of the proposed sensor more intuitively, an LED was adopted as an indicator whose luminescence was proportional to the harvested energy determined by the chemical components in the binary mixtures. As the change in the dielectric constant of the chemical compounds determines the energy-harvesting rate, the resulting output power varied from 0 mW to 7 mW depending on the chemical substances and their mixture ratios, and the brightness of the LED changed correspondingly, as shown in Figure 6b,c. Figure 6c shows the experimental results of the LED-embedded metamaterial harvesting sensor system for different chemical concentrations in the water-based binary mixtures. Hence, as the energy-harvesting rate of the sensor for toluene, chloroform, and ethanol binary mixtures decreased according to the increase in the water concentration (Figure 6a), the brightness of the LED also tended to decrease for all the chemical mixtures, and was eventually completely turned off for 100 % water concentration as the output voltage of the sensor was smaller than the turn-on voltage of the LED.

## 4. Conclusions

In summary, we presented a novel wireless chemical sensor system based on energy-harvesting metamaterials at microwave frequencies. The change in the effective dielectric constants with different chemical compounds and their mixtures led to the resonance shift of our metamaterial sensor and thus resulted in the spectral change of its energy-harvesting properties. From the simulation and experimental results, we confirmed that the proposed sensor system successfully realized wireless chemical sensing by utilizing a commercial 2.4 GHz Wi-Fi AP as a single wave source without any external power source. Moreover, as the maximum energy-harvesting rate occurred at the resonance frequency of our metamaterial device, the proposed sensor system could provide simple LED-based chemical analysis by measuring the chemical-dependent energy-harvesting rates without additional complicated measurement systems, thus, offering accessibility and simplicity for sensor applications. We believe that our results can pave the way for miniaturized wireless sensor systems, including biochemical and dielectric environment detectors.
